# A phase I clinical study of immunotherapy for advanced colorectal cancers using carcinoembryonic antigen-pulsed dendritic cells mixed with tetanus toxoid and subsequent IL-2 treatment

**DOI:** 10.1186/s12929-016-0279-7

**Published:** 2016-08-24

**Authors:** Ko-Jiunn Liu, Tsu-Yi Chao, Jang-Yang Chang, Ann-Lii Cheng, Hui-Ju Ch’ang, Woei-Yau Kao, Yu-Chen Wu, Wei-Lan Yu, Tsai-Rong Chung, Jacqueline Whang-Peng

**Affiliations:** 1 National Institute of Cancer Research, National Health Research Institutes, Tainan, Taiwan; 2 Institute of Clinical Pharmacy and Pharmaceutical Sciences, National Cheng Kung University, Tainan, Taiwan; 3 School of Medical Laboratory Science and Biotechnology, Taipei Medical University, Taipei, Taiwan; 4 Division of Hematology/Oncology, Tri-Service General Hospital, Taipei, Taiwan; 5 Department of Oncology, National Taiwan University Hospital, Taipei, Taiwan; 6 Present Address: Department of Hematology/Oncology, Taipei Medical University Shuang Ho Hospital, Taipei, Taiwan; 7 Present Address: Division of Hematology/Oncology, Department of Internal Medicine, National Cheng Kung University Hospital, College of Medicine, National Cheng Kung University, Tainan, Taiwan; 8 Present Address: Division of Hematology-Oncology, Department of Medicine, Taipei Tzu Chi Hospital, Taipei, Taiwan; 9 Present Address: Comprehensive Cancer Center, Taipei Medical University, Taipei, Taiwan

**Keywords:** Colorectal cancer, Carcinoembryonic antigen, Dendritic cell, Tetanus toxoid, Interleukin-2

## Abstract

**Background:**

To better evaluate and improve the efficacy of dendritic cell (DC)-based cancer immunotherapy, we conducted a clinical study of patients with advanced colorectal cancer using carcinoembryonic antigen (CEA)-pulsed DCs mixed with tetanus toxoid and subsequent interleukin-2 treatment. The tetanus toxoid in the vaccine preparation serves as an adjuvant and provides a non-tumor specific immune response to enhance vaccine efficacy. The aims of this study were to (1) evaluate the toxicity of this treatment, (2) observe the clinical responses of vaccinated patients, and (3) investigate the immune responses of patients against CEA before and after treatment.

**Methods:**

Twelve patients were recruited and treated in this phase I clinical study. These patients all had metastatic colorectal cancer and failed standard chemotherapy. We first subcutaneously immunized patients with metastatic colorectal cancer with 1 × 10^6^ CEA-pulsed DCs mixed with tetanus toxoid as an adjuvant. Patients received 3 successive injections with 1 × 10^6^ CEA-pulsed DCs alone. Low-dose interleukin-2 was administered subcutaneously following the final DC vaccination to boost the growth of T cells. Patients were evaluated for adverse event and clinical status. Blood samples collected before, during, and after treatment were analyzed for T cell proliferation responses against CEA.

**Results:**

No severe treatment-related side effects or toxicity was observed in patients who received the regular 4 DC vaccine injections. Two patients had stable disease and 10 patients showed disease progression. A statistically significant increase in proliferation against CEA by T cells collected after vaccination was observed in 2 of 9 patients.

**Conclusions:**

The results of this study indicate that it is feasible and safe to treat colorectal cancer patients using this protocol. An increase in the anti-CEA immune response and a clinical benefit was observed in a small fraction of patients. This treatment protocol should be further evaluated in additional colorectal cancer patients with modifications to enhance T cell responses.

**Trial registration:**

ClinicalTrials.gov (identifier NCT00154713), September 8, 2005

**Electronic supplementary material:**

The online version of this article (doi:10.1186/s12929-016-0279-7) contains supplementary material, which is available to authorized users.

## Background

Colorectal cancer (CRC) is one of the most common and deadly cancers in Taiwan and the United States [1]. Although recent developments in surgical management, chemotherapy, and biological therapy have improved the survival of early-stage CRC patients, the treatment of patients with late-stage CRC remains difficult [2]. The use of an anti-immune checkpoint antibody has revolutionized the clinical treatment of many cancers [3]. Indications for melanoma and non-small cell lung carcinoma have been approved, and the application of anti-checkpoint antibodies in other cancer types, including CRC, has been actively evaluated in the clinical setting [4–6]. However, the results of several clinical studies revealed that anti-immune checkpoint antibodies for many cancers may not function as effectively as in melanoma and lung cancers [7].

For CRC, a better therapeutic result was observed in patients with mismatch-repair deficiency than in those without this deficiency [8]. Recent reports suggested a positive relationship between clinical responses and the amounts of tumor mutation or neoantigen in patients receiving anti-immune checkpoint antibody therapy [9]. It has been hypothesized that CRC patients with mismatch-repair deficiency may accumulate a larger number of mutations and generate abundant neoantigens in their tumors, thus favoring an approach involving the reactivation of pre-existing T cells using the anti-immune checkpoint antibody. However, patients with mismatch-repair deficiency represent a very small fraction of CRC patients [10, 11]. Therefore, efforts to combine the anti-immune checkpoint antibody with other types of treatment have been proposed to increase the therapeutic efficacy for cancers displaying a lower response rate when treated with anti-immune checkpoint antibody alone [12]. One of the these approaches involves vaccinating CRC and other cancer patients with tumor-associated antigens to increase the number or diversity of T cells and followed by providing an anti-immune checkpoint antibody to strengthen or prolong T cell responses [6, 13, 14]. The dendritic cell (DC)-based cancer vaccine appears to be the most promising method for boosting the patient immune responses against tumors. DCs are the most important antigen-presenting cells in the body, and DC-based cancer immunotherapy has been extensively explored in recent years [15, 16]. Provenge (sipuleucel-T), a product based on antigen-pulsed antigen-presenting cells for the treatment of hormone-refractory prostate cancer, was approved by the FDA in 2010 [17], demonstrating the therapeutic potential of such application.

To develop an alternative therapy for patients who have failed standard chemotherapy and provide an effective adjuvant therapy for cancer patients, DC-based immunotherapy for CRC patients has been examined [18]. Elevated expression of carcinoembryonic antigen (CEA) was observed in most CRC both in the serum and tumor [19]. Although CEA is also expressed in normal colon epithelial cells, the expression level is low. Previous immunotherapies targeting CEA have shown that immune responses against CEA were elevated in patients without severe autoimmune responses [20, 21], suggesting that CEA may be useful as a tumor-associated antigen. In our previous pilot study, we pulsed patient’s autologous DCs with synthetic peptides representing the CTL epitopes on CEA. All patients tolerated the intranodal injections of DC vaccines well and no severe toxicity or autoimmunity was observed. An increase in the number of CEA-specific T cells after DC vaccination was detected in 6 of the 9 patients evaluated [20]. Two of 10 patients had stable diseases. The results of this pilot trial suggested that the vaccination procedure is feasible and safe, and that this treatment may generate or boost tumor-antigen-specific T cell responses in many patients.

Studies by others and us indicated that tumor-associated antigen-specific T cells responses can be generated in most cancer patients after DC vaccination, but these T cell responses are generally short-lived [20, 22, 23]. This may significantly limit treatment efficacy. In our previous pilot study [20], we chose to use synthetic peptides representing the CTL epitopes on CEA as the source of antigen to pulse DCs. This approach has the benefit of generating CEA peptide-specific CD8^+^ T cell responses but has the limitation that we can only vaccinate a portion of cancer patients that express a particular HLA phenotype and that CEA-specific CD4^+^ T cell responses may not be activated. Therefore, we conducted this phase I clinical study to evaluate and improve the efficacy of DC-based immunotherapy using CEA-pulsed DCs mixed with tetanus toxoid (TT) and subsequent interleukin (IL)-2 treatment. The use of whole CEA protein as the source of antigen will provide potential epitopes recognized by CD4^+^ and CD8^+^ T cells derived from patients with different HLA phenotypes. TT is a very strong recall antigen and induces a delayed-type hypersensitivity (DTH) response. We hypothesize that the local DTH response induced by TT further activates co-injected DCs and promotes their T cell-stimulating functions. Low-dose IL-2 was administered subcutaneously following DC vaccination in this study to further boost and maintain T cell growth. The results of this clinical study, including the safety evaluation, clinical status, and immune responses of patients, are reported.

## Methods

### Patient characteristics

Twelve patients, 6 from the National Taiwan University Hospital and 6 from the Tri-Service General Hospital, were enrolled in this study between 2006 and 2010. This clinical protocol was approved by the Research Ethics Committee of the National Taiwan University Hospital (protocol number 27MD02) and the Institutional Review Board of the Tri-Service General Hospital/National Defense Medical Center (protocol number 095-04-003), and further approved by the Department of Health (currently the Ministry of Health and Welfare), Taiwan. Signed informed consent was obtained from each patient before the recruitment. All patients had experienced metastasis from their primary colorectal cancer and had failed the first-line chemotherapy regimen containing CPT-11 (irinotecan) or oxaliplatin. Patients were more than 20 years old and their serum CEA levels were at least 5-fold higher than the normal limit. All patients had adequate bone marrow, liver, and renal function defined as white blood cell ≥3500/mm^3^, neutrophil ≥1500/mm^3^, lymphocyte ≥1000/mm^3^, platelet ≥100,000/mm^3^, glutamate oxaloacetate transaminase (GOT), and glutamate pyruvic transaminase (GPT) ≤5-fold of the normal range, bilirubin ≤1.5-fold of the normal range, and creatinine ≤2-fold of the normal range. Patients had appropriate immune function, defined as IgG ≥614 mg/dL, IgM ≥53 mg/dL and the DTH test showed positive results (≥5 mm in diameter). Patient performance status (PS) ranged from 0 to 2 on the ECGO scale. Patients with central nervous system metastasis, autoimmune disease, or active/chronic infection and patients who received chemotherapy, steroid, or biological treatment within 4 weeks before enrollment were excluded from this study. The complete inclusion and exclusion criteria of patient selection were listed in the Additional file 1. Selected patient characteristics are shown in Table 1.

**Table 1:** Characteristics of enrolled cancer patients

Patient	Sex	Age	PS	Metastasis site
1	Male	68	1	Liver, lung, and bone
2	Female	49	1	Liver
3	Female	48	1	Liver
4	Female	62	2	Liver and lung
5	Male	45	1	Rectum
6	Male	58	1	Liver, lung, bone and adrenal glands
7	Male	52	0	Liver
8	Male	48	1	Liver
9	Female	52	1	Liver, lung and bone
10	Male	80	1	Liver, lung, and lymph node
11	Male	65	1	Abdominal wall
12	Male	62	0	Liver

### Preparation of human DCs from peripheral blood mononuclear cells (PBMCs)

PBMCs derived from apheresis were further enriched by density gradient centrifugation in lymphocyte separation medium (Lonza, Basel, Switzerland). The PBMCs were incubated for 2 h at 37 °C in X-VIVO15 medium (Lonza) in a plastic flask, and adherent cells were cultured in X-VIVO15 medium containing 2 % heat-inactivated autologous plasma, 1000 U/mL human interleukin-4 (IL-4, GMP-grade, Strathmann Biotec AG, Hannover, Germany), and 500 U/mL granulocyte macrophage colony-stimulating factor (GM-CSF, GMP-grade, GENTAUR Belgium BVBA, Kampenhout, Belgium). On day 6, loosely attached or floating immature DCs were collected. The immature DCs were stored in the gas phase of a liquid nitrogen tank until use. No bacteria, fungus, mycoplasma, or endotoxin contamination were detected in any cell culture products. The Gram’s iodine stain method was used for bacteria contamination evaluation. The detection of bacteria and fungus contamination was further performed by a growth-based rapid microbiological method with the BacT/ALERT automatic culture system (bioMerieux SA, Marcy I’Etoile, France). The detection of mycoplasma contamination was performed using a PCR-based method (e-Myco plus mycoplasma PCR detection kit, iNtRON Biotechnology, Kyungki-Do, Korea). The endotoxin contamination was determined using a Limius Amebocyte Lysate QCL-1000 Endotoxin test (Lonza).

### Vaccine preparation and vaccination protocol

**Fig. 1:**
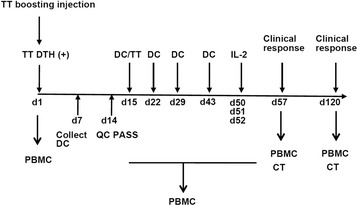
Summary of treatment procedures in this study. Enrolled patients were injected with tetanus toxoid (TT), and those with positive TT DTH responses were subjected to leukopheresis to generate DCs for vaccine preparation. Cells were cultured in GM-CSF and IL-4 to generate immature DCs (day 1, d1). After 6 days, immature DCs were collected and checked for compliance with QC requirements (d7). DC preparations passed for QC were thawed on d14 and pulsed with rhCEA for 3 h and then stimulated with TNF-α and INF-γ for 16 h to generated mature DCs. Next, 1 × 10^6^ rhCEA-pulsed DCs were injected subcutaneously into patients on days 15, 22, 29, and 43. For the first injection, the DC vaccine was mixed with TT. One week after the last DC vaccine injection (d50), patients were subcutaneously injected with IL-2 for 3 days (d51–d53). PBMCs were collected before, during, and after DC vaccination for immune responses analysis. Patients were subjected to CT examination for clinical responses 6 weeks after the first DC vaccine injection (d57), and every 2 months afterwards until disease progression

Thawed immature DCs (3 × 10^6^) were suspended in 1 mL of X-VIVO15 medium and cultured with 25 μg/mL of recombinant human CEA (rhCEA, Protein Sciences Corp., Meriden, CT, USA) at 37 °C. After 3 h, rhCEA-pulsed DCs were collected and matured by culturing the cells in X-VIVO15 medium containing 2 % heat-inactivated autologous plasma and 1000 U/mL tumor necrosis factor-α (TNF-α, CELL-GRO, CellGenix, Freiburg im Breisgau, Germany), recombinant human interferon-gamma (IFN-γ, GMP-grade, GENTAUR Belgium BVBA), and human IL-4 (GMP-grade, Strathmann) in a T25 flask at 37 °C for 18 h. The cells were collected and used as rhCEA-pulsed, matured DCs. The procedure of pulsing DCs with rhCEA was modified from a previous study [24]. In this study, we did not actually verify the efficiency of CEA presentation by DCs pulsed with rhCEA. Collected DCs were washed five times with normal saline, and 1.5 × 10^6^ DCs were suspended in 0.3 mL normal saline supplemented with 1 % heat-inactivated autologous plasma. To remove cell clusters, the cell suspension was slowly passed through a 25-gauge needle. The cell suspension was then injected subcutaneously near one inguinal lymph node of the patient. For the first DC vaccine injection, the cell suspension was mixed with diluted tetanus toxoid (0.04 U in 0.1 mL, Adimmune Corp., Taichung, Taiwan) before injection. Patients were vaccinated once per week for 3 weeks followed by a boost injection 2 weeks later. IL-2 (Proleukin, Chiron, Emeryville, CA, USA) was injected subcutaneously (5 × 10^6^ IU/m^2^, twice/day × 3 days) 1 week after the fourth DC vaccination. Whole blood (30 mL) was collected from the patients 2 weeks after the first and last injections. PBMCs were purified and cryopreserved to evaluate the immune responses against CEA. Patients received the first CT examination 6 weeks after the first DC vaccination and every 2 months afterward. Patients showing stable or better clinical responses received 1 boost injection of DC vaccine every 2 months until disease progression. The schedule of DC vaccination and treatment of this study is shown in Fig. 1.

### Flow cytometry analysis of immature DCs

Immature DCs were stained with different fluorescence-labeled monoclonal antibodies (mAbs) and then analyzed using a flow cytometer (FACSCalibur; BD Biosciences, Franklin Lakes, NJ, USA). The mAbs used in the study included: FITC-anti-HLA-DR (Beckman Coulter, Brea, CA, USA), phycoerythrin-anti-CD86 (Beckman Coulter), FITC-anti-CD80 (Immunotech, Marseille Cedex, France), phycoerythrin-anti-CD86 (Beckman Coulter), phycoerythrin-anti-CD83 (Immunotech, Marseille Cedex, France), phycoerythrin-anti-CD14 (BD Biosciences), and FITC-anti-CD40 (Biolegend, San Diego, CA, USA). Isotype-matched control mAbs were obtained from Biolegend. Results were expressed as the percentage of cells stained positive for a given mAb.

### T-cell proliferation assay

PBMCs collected before and after vaccination were thawed at the same time for immunologic analysis. Three replicates of 1 × 10^5^ PBMCs were cultured in 96-well culture plates for 5 days in RPMI-1640 supplemented with 5 % human type-AB serum (Lonza) in the presence of 0 (PBS), 5, 10, 25, or 50 μg/mL rhCEA protein (Fitzgerald, Fitzgerald Industries International. Acton, MA, USA). Cellular proliferation was determined using a bromodeoxyuridine (BrdU) incorporation enzyme-linked immunosorbent assay (ELISA) kit (Roche Diagnostics GmbH, Mannheim, Germany). T cell proliferation status was expressed as the OD value measured at 450 nm (reference wavelength 650 nm). The raw data of T cell proliferation assay was provided in the Additional file 2.

### Toxicity and clinical evaluation

Toxicity grading was conducted and recorded according to National Cancer Institute Common Terminology Criteria for Adverse Events (CTCAE) v4.0. Clinical tumor responses were defined following the Response Evaluation Criteria in Solid Tumors v1.0.

### Statistical analysis

Data are presented as mean ± SD and differences between means were analyzed with the Student’s *T* test using Microsoft Excel software (Redmond, WA, USA). Differences were considered significant at *P* < 0.05.

## Results

### Patients and vaccine preparation

Twelve patients were enrolled and treated in this clinical study (Table 1). All patients recruited showed proper bone marrow, liver, kidney, and immune functions and fit the inclusion and exclusion criteria of this study. Selected baseline clinical data of these patients are listed in Tables 2 and 3. These patients all displayed positive DTH responses against tetanus toxoid after the boosting injection and had adequate serum IgG and IgM levels (Table 3), suggesting proper basic immune functions after previous chemotherapy. The schedule of DC vaccination in this study is shown in Fig. 1. The DCs generated from patients’ mononuclear cells displayed proper surface markers (more than 80 % positive for HLA-DR, CD86, and CD40) and met the release criteria of the in-process cell products (Table 4). For each DC vaccine preparation, the supernatant from the day 6 DC culture and supernatant after the last washing were examined for contamination with endotoxin, mycoplasma, fungus, and bacteria. All samples analyzed were negative for microorganism contamination and the endotoxin level was always below 0.15 endotoxin U/mL. These results indicate that the quality of our vaccine product is adequate.

**Table 2:** Baseline clinical data (1)

Patient	CEA	Leukocyte	Neutrophil	Lymphocyte	Platelets
1	82.3	4460	3010	1057	310,000
2	323.3	7310	5695	1301	240,000
3	53.5	5280	3559	1362	217,000
4	645.7	7130	4442	2081	207,000
5	391.2	6100	4111	1482	250,000
6	291.7	7700	5328	1371	279,000
7	1388.1	13700	9410	1980	253,000
8	849	7300	4679	1365	229,000
9	500.6	8730	6590	1090	275,000
10	60.3	5970	3647	1528	146,000
11	298.8	9740	7305	1705	350,000
12	28.7	5330	2740	2180	205,000

**Table 3:** Baseline clinical data (2)

Patient	GOT/GPT	Bilirubin	Creatinine	IgG IgM	DTH skin test
1	26/40	0.89	1	IgG (1170) IgM (58.4)	16 mm × 15 mm
2	47/38	1.15	0.8	IgG (1330) IgM (79.8)	10 mm × 7 mm
3	19/14	1.0	0.8	IgG (1530) IgM (54.9)	5 mm × 5 mm
4	38/19	0.95	0.7	IgG (1890) IgM (56.2)	30 mm × 30 mm
5	20/23	0.7	1.1	IgG (1320) IgM (96)	6 mm × 6 mm
6	22/9	0.9	1.2	IgG (1560) IgM (83)	8 mm × 11 mm
7	43/34	0.4	1.1	IgG (821) IgM (56)	8 mm × 10 mm
8	50/41	1.4	0.8	IgG (1500) IgM (64)	10 mm × 8 mm
9	39/15	0.4	0.5	IgG (1150) IgM (86)	12 mm × 10 mm
10	40/24	0.7	0.8	IgG (1630) IgM (76)	11 mm × 11 mm
11	19/12	0.53	0.9	IgG (1580) IgM (83.9)	7 mm × 9 mm
12	25/24	1.5	0.9	IgG (1270) IgM (93.2)	12 mm × 12 mm

**Table 4:** Surface marker expression of DC 6 days after culture from PBMCs

Marker	HLA-DR	CD86	CD40
Patient			
1	93.39^a^	96.47	98.22
2	95.4	85.36	99.41
3	83.14	94.17	95.51
4	95.2	94.98	96.64
5	97.92	93.64	98.96
6	95.98	87.72	94.75
7	84.4	93.08	97.24
8	86.25	95.35	99.18
9	94.03	96.22	94.54
10	90.67	93.29	97.71
11	89.52	97.43	98.25
12	95.9	93.76	95.97
Mean ± SD	91.8 ± 4.96	93.5 ± 5.34	97.2 ± 1.71

^a^Results were expressed as the percentage of cells stained positive for a given mAb

### Adverse events and autoimmune profiles

The primary endpoint of this study was the safety of the treatment procedure. No severe treatment-related side effects or toxicity was observed in patients who received the regular 4 DC vaccine injections. However, patients 2 and 8 showed early disease progression during the treatment period and only received 2 and 3 DC vaccinations, respectively. Some evaluation data were not available from these 2 patients because of their early withdrawal from the study. The remaining 10 patients received at least 4 standard DC vaccinations and subsequent IL-2 administration. A comparison of liver and kidney functions of the patients before, during, and after DC vaccination is shown in Table 5. Patients 4 and 8 showed a more substantial increase in GOT during the study (day 36) and were subsequently removed from the study because of disease progression. Several other patients showed minor elevations in GOT/GPT, but the level remained within the initial patient selection criteria (5-fold of normal value). Patient 12 showed a minor increase in bilirubin on day 36, but returned to the initial level on day 57. Patient 8 showed a substantial increase in bilirubin on day 36 and was subsequently removed from the study because of disease progression. Patient 6 showed an increase in creatinine on days 36 and 57, but the level remained within the initial patient selection criteria (2-fold of normal value). These detection values were within the limit of grade II toxicity.

**Table 5:** Comparison of liver and kidney function of patients before, during, and after DC vaccination

Patient	Liver function						Kidney function		
	GOT/GPT			Bilirubin			Creatinine		
	Day 0 (before)	Day 36 (during)	Day 57 (after)	Day 0 (before)	Day 36 (during)	Day 57 (after)	Day 0 (before)	Day 36 (during)	Day 57 (after)
1	26/40	40/33	47/37	0.89	1.04	1.35	1.0	1.1	1.0
2	47/38	off^a^	off	1.15	off	off	0.8	off	off
3	19/14	27/26	54/84	1.0	0.68	0.65	0.8	0.7	0.7
4	38/19	94/37	off	0.95	1.01	off	0.7	0.8	off
5	20/23	17/17	20/39	0.7	1.1	0.5	1.1	1.2	1.0
6	22/9	26/11	22/13	0.9	0.8	1.0	1.2	2.2	2.3
7	24/39	52/55	47/46	0.4	1.2	1.0	1.0	0.9	0.8
8	50/41	132/61	off	1.4	35.5	off	0.8	0.8	off
9	39/15	29/14	41/28	0.4	0.4	0.3	0.5	0.5	0.4
10	32/17	53/30	49/48	0.9	0.8	0.9	0.9	0.8	0.7
11	19/12	20/11	23/14	0.53	0.3	0.33	0.9	0.8	0.8
12	25/24	41/45	28/30	1.5	2.25	1.53	0.9	1.0	1.0

^a^“off” indicates samples not available for analysis because of withdrawal or termination of enrolled patients from this study

Autoimmune factors, including anti-nuclear antibody, rheumatoid factor, and thyroglobulin antibody, were not substantially increased after treatment in most patients. However, final data were unavailable from three patients because of disease progression (Table 6). Patient 9 showed a higher level of rheumatoid factor before treatment, and the level slightly decreased during and after the 4 DC vaccine injections. Patient 12 showed a higher level of thyroglobulin antibody before treatment, and the level decreased during and after the 4 DC vaccine injections. Overall, we observed no significant changes in the autoimmune profiles of the patients.

**Table 6:** Comparison of autoimmune profiles before, during, and after vaccination

Patient	Antinuclear antibody			Rheumatoid factor			Thyroglobulin antibody		
	Day 0 (before)	Day 36 (during)	Day 57 (after)	Day 0 (before)	Day 36 (during)	Day 57 (after)	Day 0 (before)	Day 36 (during)	Day 57 (after)
1	1:40	1:40	1:40	<20	<20	<20	1:40	1:40	1:40
2	1:40	off^a^	off	<20	off	off	1.7	off	off
3	1:40	1:40	1:40	<20	<20	<20	1:40	6.21	1:40
4	1:40	1:80	off	<20	<20	off	1:40	1:40	off
5	(−)	(−)	(−)^b^	<20	22.9	23.3	1:80	1:80	<1:10
6	(−)	(−)	(−)	<20	<20	<20	1:80	<1:10	<1:10
7	(−)	(−)	(−)	<20	<20	<20	1:40	1:80	1:40
8	(−)	(−)	off	<20	<20	off	1:80	1:40	off
9	(−)	(−)	(−)	60.6	59.5	44.2	1:160	1:80	<1:10
10	1:640	1:>1280	1:>1280	<20	20.5	21.4	1:40	<1:10	1:40
11	1:40	1:40	1:40	<20	<20	<20	5.11	3.09	7.81
12	1:40	1:40	1:40	<20	<20	<20	14.2	0.71	1.14

“off” indicates samples were not available for analysis due to withdraw or termination of enrolled patients from this study

^a^“off” indicates samples not available for analysis because of withdrawal or termination of enrolled patients from this study

^b^(−): not detected

In the evaluation of other side effects and toxicity (Table 7), patient 1 had grade III diarrhea before treatment and the symptom persisted during the trial period. Patient 2 had grade III diarrhea on day 22, which was resolved soon after. Patient 6 had grade II creatinine elevation, likely because of bone metastasis of his disease. Some other minor grade I or II side effects and toxicities were occasionally observed, but were not persistent or severe. There was no significant or rapid body weight loss in patients following DC vaccine injections. Taken together, these results indicate that the injection of rhCEA-pulsed DCs into late-stage colorectal cancer patients using our vaccination process is generally safe.

**Table 7:** Frequency and grade of adverse events of the 12 patients

Grade^a^	I	II	III	IV	V
Adverse event					
Local pain	3/12^b^	2/12			
Local swelling		1/12			
Fever	5/12	2/12			
Skin rash/itching/skin desquamation	3/12	2/12			
Myalgia	1/12				
Bilirubin	4/12	1/12	2/12	1/12	
GPT	9/12	2/12			
GOT	9/12	2/12	1/12		
Hemorrhage (UGI^c^)		1/12			
Diarrhea/Mucocitis	3/12	1/12	2/12		
Creatinine	1/12	1/12			
Allergy	1/12	1/12			
Anemia	1/12	1/12			
Short of breath	2/12				
Sweating	1/12				
Chillness	1/12				
Leg edema	1/12				

^a^Adverse event was determined using CTCAE version 4.0

^b^“3/12” indicates that 3 of 12 patients had at least one episode of a particular adverse event

^c^UGI indicates upper gastrointestinal

### Clinical and immune responses

In the observation of clinical response of these patients, patients 5 and 12 showed stable disease after standard vaccination (Table 8). Therefore, these 2 patients received 2 and 1 boosting injection(s), respectively, until disease progression. The total duration of disease stable period was 105 and 98 days for patients 5 and 12 (Table 8). The other 10 patients had disease progression either during the treatment period or at the first evaluation time point after the treatment procedure. The overall rate of patients with clinical benefit was 16.7 % for all 12 enrolled patients and 20 % for the 10 patients completing the standard 4 DC vaccination protocol.

**Table 8:** Summary of DC vaccination and clinical responses

Patient	DC injections Regular (boosting)	Best response (duration)
1	4	PD^a^
2	2	PD
3	4	PD
4	4	PD
5	4(2)	SD^b^ (105 days)
6	4	PD
7	4	PD
8	3	PD
9	4	PD
10	4	PD
11	4	PD
12	4(1)	SD (98 days)

^a^PD: progressive disease

^b^SD: stable disease

**Fig. 2:**
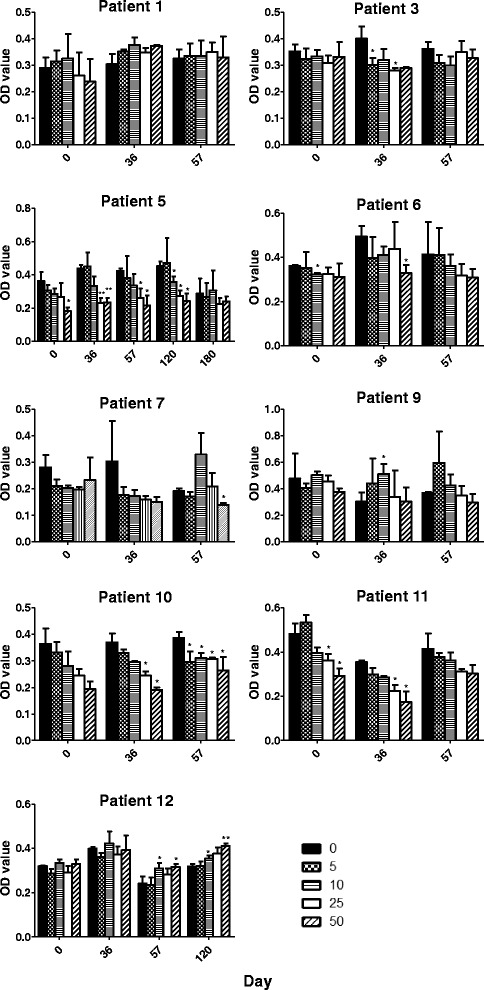
Proliferation against rhCEA by PBMCs collected before, during, and after DC vaccination from different patients. PBMCs collected before (day 0), during, and after DC vaccination (days 36, 57, 120, and 180) were cultured in 96-well culture plates for 5 days in the presence of 0 (PBS), 5, 10, 25, or 50 μg/mL rhCEA. Cellular proliferation was determined using a BrdU incorporation ELISA. T cell proliferation status was expressed as the OD value measured at 450 nm (reference wavelength 650 nm). The OD value obtained from culture with different concentrations of rhCEA was compared to that of culture with PBS control. *p < 0.05, **p < 0.001

For the investigation of immune responses after DC vaccination, PBMCs collected before and after DC vaccination were thawed at the same time for immunological analysis. Cells were cultured with 5–50 μg/mL of rhCEA for 5 days and then cellular proliferation was determined. Blood samples from 9 patients were analyzed for T cell responses against rhCEA; while blood samples after treatment were not available from 3 patients because of early disease progression and patients’ reluctance to blood drawing, and were therefore not examined for T cell responses. As shown in Fig. 2, most T cell responses against rhCEA were not strong. In some of the patients, addition of rhCEA appears to induce cell death as reflexed by the significant reduction of BrdU incorporation. Patients 5 and 12 showed stable disease after standard vaccination and therefore received 2 and 1 boosting injection(s), respectively. The proliferation of T cells was determined with additional PBMCs collected on day 180 (patient 5) and day120 (patients 5 and 12). A statistically significant increase in the T cell proliferation against rhCEA (10 and 50 μg/mL) was observed in patient 12, but not in patient 5, with PBMCs collected on day 57 and day 120, suggesting the presence of a persistent T cell response. A temporary significant increase in T cell proliferation against rhCEA (10 μg/mL) was observed in patient 9 with PBMCs collected on day 36.

## Discussion

The results of this study indicated that the strategy of combining the DC vaccine with TT followed by low-dose IL-2 injection is generally safe for patients with late-stage CRC. No severe treatment-related toxicity was observed in any evaluable patients after DC vaccination. The vital signs of all patients were stable after vaccination. As described above, patients 2 and 8 showed early disease progression during the treatment period and only received 2 and 3 DC vaccinations, respectively. Patient 4 showed disease progression shortly after completion of the treatment. Some evaluation data were not available from these patients because of early withdrawal from the study. Patient 9 showed a higher level of rheumatoid factor before treatment, and the level slightly decreased during and after the 4 DC vaccine injections. Patient 12 showed a higher level of thyroglobulin antibody before treatment, and the level decreased during and after the 4 DC vaccine injections. Patient 1 had grade III diarrhea before treatment, and the symptom persisted during the trial period. Patient 2 had grade III diarrhea on day 22, but this was resolved soon after. Patient 6 had grade II creatinine elevation, likely because of the bone metastasis of his disease. Some other minor grade I or II side effects or toxicities were occasionally observed, but were not persistent or severe.

In this study, we found that vaccination of DCs pulsed with rhCEA is feasible and safe in the laboratory and clinical setting. Such vaccination may induce T cell responses in certain cancer patients. However, these responses were moderate at best. In patient 12 with stable disease, the proliferation of T cells against rhCEA was detected at 57 and 120 days after initial injection. However, such increase in the T cell proliferation was not observed in patient 5 who also had stable disease after treatment. These results suggest that T cell proliferation against CEA may not be the most adequate method to determine the immune status in patients of this study, or the clinical benefit may result from immune responses against tumor-associated antigens other than CEA. Addition of rhCEA resulted in death of PBMCs from several patients in the T cell proliferation assay. We can not rule out the possibility that this is due to an antigen-induced apoptosis of T cells.

Attempts to stimulate or maintain a prolonged T cell response in vaccinated patients have been explored in many studies. These efforts include improvement of DC stimulation [25, 26], pre-conditioning of the vaccination site [27], and measures to maintain a long-lasting T cell response with cytokines or other biological agents [28]. We previously reported in animal models a strategy for pulsing DCs with CD40 ligand-transfected tumor cells and pre-conditioning with MIP-3α-transfected tumor cells [29, 30]. Both approaches resulted in a better immune response against the tumor and effectively suppressed tumor growth and metastasis. This current study focused on the incorporation of the recall antigen TT in vaccine preparation and supplement of cytokines such as IL-2 after vaccination to increase the longevity of T cell responses. A recent study using TT to pre-condition the vaccination site of DCs pulsed with *Cytomegalovirus* phosphoprotein 65 RNA to treat glioblastoma patients also demonstrated an increase in the migration of DCs to draining lymph node and improved clinical outcomes [31]. Alternatively, strategies for removing or suppressing regulatory T cell activity in vivo were shown to enhance the T cell responses [28]. Another attractive strategy is to isolate T cells from patients after vaccination, expand and activate these T cells to a large quantity in vitro, and infuse the activated T cells back into the patients [32, 33]. The expansion of T cells in vitro may potentially bypass the negative influence of regulatory T cells in the body. In addition, repeated infusions of a large number of tumor-associated antigen-specific T cells would be possible using this approach. Thus, a combination of different immunotherapy strategies, DC vaccination, and adoptive T cell therapy, may increase the efficacy of cancer treatment [18, 34]. We are currently investigating the potential of such combined immunotherapy.

## Conclusions

The results of this clinical study were compatible to the safety data and clinical observation reported for other cancers involving DC-based immunotherapy [15, 16]. Although the results of our clinical study are encouraging, most patients still showed disease progression during or after the DC vaccination. Additionally, these 12 patients were in the advanced disease stage and had failed all available treatments before entering this study. These results strengthen the view that DC-based immunotherapy should be performed in patients with early disease status or combined with other clinical interventions such as anti-immune checkpoint antibodies or adoptive T cell therapies to obtain better treatment outcomes.

## Additional files

Additional file 1: The complete inclusion and exclusion criteria of patient selection. (DOC 30 kb) Additional file 2: The results of T cell proliferation assay. (XLS 93 kb)

## Abbreviations

BrdU: Bromodeoxyuridine
CRC: Colorectal cancer
CTCAE: Common Terminology Criteria for Adverse Events
CTL: Cytotoxic T lymphocyte
DC: Dendritic cell
DTH: Delayed-type hypersensitivity
ECOG: European cooperative oncology group
ELISA: Enzyme-linked immunosorbent assay
FITC: Fluorescein isothiocyanate
GM-CSF: Granulocyte macrophage colony-stimulating factor
GMP: Good manufacturing practice
GOT: Glutamate oxaloacetate transaminase
GPT: Glutamate pyruvic transaminase
HLA: Human leukocyte antigen
IL-2: Interleukin-2
IFN: Interferon
Ig: Immunoglobulin
mAb: Monoclonal antibody
PBMC: Peripheral blood mononuclear cell
PD: Progressive disease
rhCEA: Recombinant human carcinoembryonic antigen
SD: Stable disease
Th1: T helper 1
TNF: Tumor necrosis factor
TT: Tetanus toxoid
U: Unit
