# Supplementary material for: A phase I clinical study of immunotherapy for advanced colorectal cancers using carcinoembryonic antigen-pulsed dendritic cells mixed with tetanus toxoid and subsequent IL-2 treatment
Source: J Biomed Sci. 2016 Aug 24;23(1):64. doi: 10.1186/s12929-016-0279-7 (PMC4997699; doi:10.1186/s12929-016-0279-7)
Supplement: Additional file 1: — The complete inclusion and exclusion criteria of patient selection. (DOC 30 kb) [file 12929_2016_279_MOESM1_ESM.doc]

Additional file 1. The inclusion and exclusion criteria of patient selection

(a) Inclusion criteria

- Patients must have metastatic colorectal cancer.
- Patients must have at least one measureable lesion.
- Patients’ serum level of CEA must be higher than 5 times of the normal value
- Patients’ disease must have failed chemotherapy with 5FU, CPT-11 or oxaliplatin. Patients who are unsuitable or refuse chemotherapy will be considered eligible for this trial.
- Patients’ age must be 20 or greater.
- Patients’ estimated life expectancy is more than 3 months.
- Patient performance status is between ECOG 0-2.
- Patients must have adequate bone marrow function, defined as WBC ≥ 3500/mm^3^, neutrophil ≥ 1500/mm^3^, lymphocyte ≥ 1,000/mm^3^, and platelet ≥ 100,000/mm^3^.
- Patients must have adequate immune status, defined as IgG ≥ 614 mg/dl, IgM ≥ 53 mg/dl), and have a positive delayed-type hypersensitivity reaction against tetanus toxoid (≥5 mm in diameter).
- Patients must have adequate liver and renal function, defined as serum alanine transaminase and aspartate transaminase ≤ 5 times normal limit, bilirubin ≤ 1.5 times normal limit, and creatinine ≤ 2 times normal limit.
- All patients should have documentation of a negative result of penicillin test.
- Women or men of reproductive potential may not participate unless they have agreed to use an effective contraceptive method.
- All patients must be informed of the investigational nature of this study and must sign and give written informed consent.

(b) Exclusion Criteria

- Patients who have central nervous system metastasis
- Patients who have active acute or chronic infection (at the discretion of the investigator).
- Pregnant or breast-nursing women
- Patients who have active cardiac disease requiring therapy for failure, angina, arrythmia, or infarction within the preceding 6 months (exception: any patient whose cardiac failure is compensated on medications)
- Patients who have asthma
- Patients who have autoimmune disease such as inflammatory bowel disease, lupus erythematosus, ankylosing spondylitis, scleroderma, and multiple sclerosis
- Patients who have serious concomitant systemic disorders incompatible with the study (at the discretion of the investigator)
- Patients who have other prior or concurrent malignancy except for in-situ-carcinoma of cervix or adequately treated basal cell carcinoma of skin.
- Patients who received chemotherapy, steroid or biologic treatment within 4 weeks prior to enrollment
